# Nanobody-Based Probes for Subcellular Protein Identification and Visualization

**DOI:** 10.3389/fncel.2020.573278

**Published:** 2020-11-02

**Authors:** Marit A. de Beer, Ben N. G. Giepmans

**Affiliations:** Department of Biomedical Sciences of Cells and Systems, University of Groningen, University Medical Center Groningen, Groningen, Netherlands

**Keywords:** nanobody, chromobody, fluobody, probes, light microscopy, super-resolution microscopy, electron microscopy, tagging

## Abstract

Understanding how building blocks of life contribute to physiology is greatly aided by protein identification and cellular localization. The two main labeling approaches developed over the past decades are labeling with antibodies such as immunoglobulin G (IgGs) or use of genetically encoded tags such as fluorescent proteins. However, IgGs are large proteins (150 kDa), which limits penetration depth and uncertainty of target position caused by up to ∼25 nm distance of the label created by the chosen targeting approach. Additionally, IgGs cannot be easily recombinantly modulated and engineered as part of fusion proteins because they consist of multiple independent translated chains. In the last decade single domain antigen binding proteins are being explored in bioscience as a tool in revealing molecular identity and localization to overcome limitations by IgGs. These nanobodies have several potential benefits over routine applications. Because of their small size (15 kDa), nanobodies better penetrate during labeling procedures and improve resolution. Moreover, nanobodies cDNA can easily be fused with other cDNA. Multidomain proteins can thus be easily engineered consisting of domains for targeting (nanobodies) and visualization by fluorescence microscopy (fluorescent proteins) or electron microscopy (based on certain enzymes). Additional modules for e.g., purification are also easily added. These nanobody-based probes can be applied in cells for live-cell endogenous protein detection or may be purified prior to use on molecules, cells or tissues. Here, we present the current state of nanobody-based probes and their implementation in microscopy, including pitfalls and potential future opportunities.

## Introduction

Defining protein identity and visualizing protein localization is fundamental in biology. Uncovering dynamics of protein localization and function were boosted when green fluorescent protein (GFP) and other fluorescent proteins (FPs) were developed and used to tag proteins of interest (Tsien, 1998; Giepmans et al., 2006; Rodriguez et al., 2017). Advantages of these chimeric fusion proteins include the lack of distance between protein of interest and label, thereby improving the resolution, as well as the specificity of labeling derived from the genetic fusion. Disadvantages include modification of the target protein, with the consequence that unmodified endogenous proteins cannot be studied (Giepmans et al., 2006). To detect endogenous proteins, immunolabeling using antibodies (immunoglobulins, mostly of the IgG isotype; IgGs) conjugated with small fluorophores are typically applied. However, for intracellular targeting IgGs require plasma membrane permeabilization leading to a damaged ultrastructure (Schnell et al., 2012). Furthermore, IgGs are large (∼150 kDa; ∼14 nm long; Table 1). This may result in a distance greater than 25 nm between target and label in indirect conventional immunolabeling, the so-called linkage error (Muyldermans, 2013; Mikhaylova et al., 2015). In addition, IgGs are multidomain proteins which require post-translational modifications (Muyldermans, 2013) and therefore preclude routine controlled genetic modification and modular expression in conjunction with e.g., GFP.

**TABLE 1:** Overview of different probes used in microscopy.

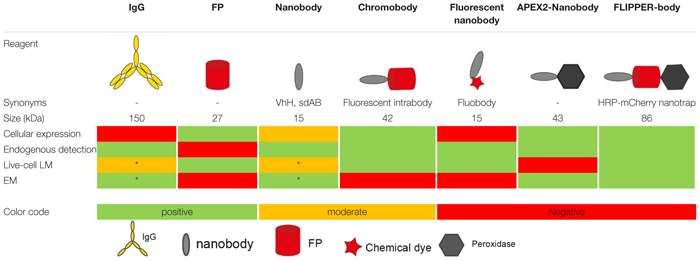

*Requires secondary labeling step.

Nanobodies are single variable domains of heavy-chain only antibodies(hcAB) derived from *Camelidae* species (Hamers-Casterman et al., 1993; Muyldermans, 2013; Helma et al., 2015; Van Audenhove and Gettemans, 2016), but do not compromise in the binding-affinity compared to IgGs, due to its complementarity-determining region (CDR) organization (Muyldermans et al., 2001; Muyldermans, 2013; Beghein and Gettemans, 2017). Nanobodies have been explored since 2006 as labeling tools in light microscopy (LM) (Rothbauer et al., 2006), because of the several potential advantages of nanobodies over other labeling techniques. Nanobody-mediated targeting for protein identification is more precise than IgG targeting, as nanobodies are only ∼15 kDa with a diameter of 2–3 nm (Table 1) and can be encoded by a relative short stretch single cDNA of 360 base pairs (Van Audenhove and Gettemans, 2016; Traenkle and Rothbauer, 2017; Carrington et al., 2019). This cDNA can genetically be fused to FPs cDNAs for intracellular (live-cell) imaging or tags can be added for purification and chemical modifications. Like IgGs, customized nanobodies can be created against a protein of interest and the cDNA can be shared free of charge, as opposed to IgGs (Zuo et al., 2017; McMahon et al., 2018). Here, an overview is given about the past and potential future of nanobody application in microscopy.

## Nanobodies in Light Microscopy

**FIGURE 1:**
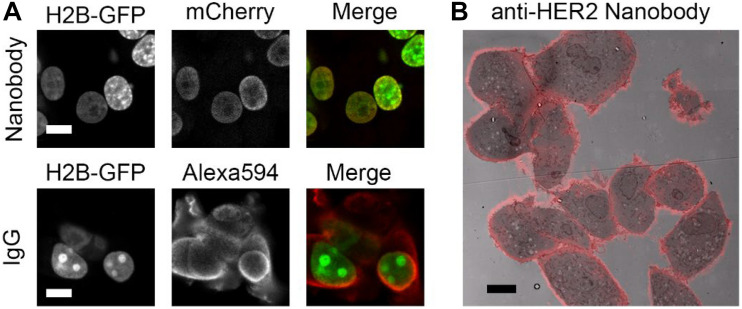
Nanobodies improve penetration and detect endogenous proteins. **(A)** Anti-GFP nanobody labeling (mCherry and peroxidase fused) and IgG labeling in H2B-GFP expressing cells. Cells permeabilized for 5 min with 0.1% Triton before labeling. Nanobodies and primary and secondary antibodies incubated for 1 h each. Note the colozalization between GFP and mCherry (nanobody), most prominently in the low-expressing cells, while Alexa Fluor 594 (IgG) mainly localizes in the cytoplasm. **(B)** High HER2 expressing cells, SkBr3, labeled with nanobodies targeting HER2. Overlay of nanobody fluoresence and EM image. Note the positive labeling at cell-cell contact sites. Bars: 10 μm. Reproduced from De Beer et al. (2018), http://creativecommons.org/licenses/by/4.0/.

Nanobodies (see Box 1 for terminology) can be expressed in cells conjugated to a detection module (like GFP) to target endogenous intracellular proteins, or they can be expressed, purified and then applied in immunolabeling resembling traditional immunofluorescence approach. Conventional immunolabeling is performed using IgGs, but for an improved penetration nanobodies can be used as an alternative (Fang et al., 2018). The improved penetration of nanobodies is illustrated in nuclear labeling of anti-GFP labeling targeting Histone2B (H2B)-GFP. Note that, in an equal labeling time, the nanobodies are colocalizing in the nucleus with the GFP, whereas the IgGs are mainly localized in the cytoplasm (Figure 1A). Though the anti-GFP is the best characterized and most used nanobody to date (Table 2), nanobodies are also used for visualizing endogenous proteins (Figure 1B and Table 2). Finally, nanobodies can be applied for live-cell imaging, both as purified proteins typically targeting extracellular antigens or being expressed from its introduced cDNA and targeted to intracellular antigens (Rothbauer et al., 2006; Ries et al., 2012).

**Box 1. Nanobody terminology Nanobody**

Synonyms: single domain antibody (sdAB), variable domain of heavy-chain only antibody (VhH), nAbs. Small antigen binding protein, derived from heavy-chain only antibodies (hcAB). These are produced in cell culture or in bacteria. **Intrabody** The cDNA of this probe is expressed in the cells for intracellular antigen targeting. **Chromobody** Synonyms: Fluobody, fluorescent nanobody. Genetic fusion of intrabody and fluorescent protein. Direct visualization with microscopy is possible. **Labeled nanobodies** Synonyms: fluobody, fluorescent nanobody. Nanobodies protein purified and tagged in vitro with e.g., a chemical dye.

**TABLE 2:** Nanobody implemented in microscopy – An overview of targets that have been visualized using nanobodies and microscopy.

**Target**	**Delivered as**	**Technique**	**References**
Actin	cDNA or Protein	LM / SR	Rocchetti et al., 2014; Panza et al., 2015; Plessner et al., 2015; Moutel et al., 2016; Periz et al., 2017; Wegner et al., 2017; Abdellatif et al., 2019; Tosetti et al., 2019
Active β2-Ars	cDNA	LM	Irannejad et al., 2013
Alexandrium Minutum	cDNA	LM	Mazzega et al., 2019
ALFA-tag	cDNA or Protein	LM / SR	Gotzke et al., 2019
AMIGO-1	cDNA	LM	Dong et al., 2019
Amyloid β	Protein	LM	Li et al., 2016
Arabidopsis Thaliana	Protein	EM	De Meyer et al., 2014
ARTC2	Protein	LM	Bannas et al., 2015a,b
ATP7B	cDNA	LM	Huang et al., 2014
bacteriophage p2	Protein	EM	De Haard et al., 2005
BC2-tag	Protein	LM / SR	Braun et al., 2016; Virant et al., 2018
β-catenin	cDNA or Protein	LM	Traenkle et al., 2015; Hebbrecht et al., 2020
BFP	Protein	SR	Sograte-Idrissi et al., 2019
CapG	cDNA	LM	De Clercq et al., 2013; Van Audenhove et al., 2013; Van Impe et al., 2008
CD11b	Protein	LM / CLEM	Rashidian et al., 2015; Duarte et al., 2016; Fang et al., 2018; Wöll et al., 2018; Cheloha et al., 2019
CEA	Protein	LM	Hafian et al., 2014; Ramos-Gomes et al., 2018
*C.Jejuni*	Protein	LM	Riazi et al., 2013
Clostridium Difficile toxin	Protein	LM	Pizzo et al., 2018
Cortactin	cDNA	LM	Van Audenhove et al., 2014, 2015; Bertier et al., 2018; Hebbrecht et al., 2020
Ebolavirus	Protein	LM	Darling et al., 2017; Sherwood and Hayhurst, 2019
EGFR	Protein	LM / CLEM	Iqbal et al., 2010a; Oliveira et al., 2012; Van De Water et al., 2012; Zarschler et al., 2014; Kooijmans et al., 2016; Krüwel et al., 2016; Van Driel et al., 2016; De Beer et al., 2018; Beltrán Hernández et al., 2019; Karges et al., 2019
Eps15	cDNA	LM	Traub, 2019
Extracellular vesicles	Protein	EM	Popovic et al., 2018
Fascin	cDNA	LM	Van Audenhove et al., 2015; Gross et al., 2016
FGFR1	cDNA	LM	Monegal et al., 2009
γ-H2Ax	cDNA	LM	Rajan et al., 2015
GPCR	cDNA	SR	Sungkaworn et al., 2017
Gelsolin	cDNA	LM	Van Impe et al., 2008; Van Den Abbeele et al., 2010; De Clercq et al., 2013; Van Audenhove et al., 2013
Gephyrin	cDNA	LM	Dong et al., 2019
GFAP	Protein	LM / CLEM	Li et al., 2012; Fang et al., 2018
GFP / YFP	cDNA or Protein	LM / SR/CLEM	Rothbauer et al., 2006, 2008; Bazl et al., 2007; Schornack et al., 2009; Kirchhofer et al., 2010; Caussinus et al., 2011; Casas-Delucchi et al., 2012; Han et al., 2012; Li et al., 2012; Pellis et al., 2012; Ries et al., 2012; Herce et al., 2013, 2017; Szymborska et al., 2013; Truan et al., 2013; Bleck et al., 2014; Wang et al., 2014; Ariotti et al., 2015, 2017, 2018; Finnigan et al., 2015; Harmansa et al., 2015, 2017; Kamiyama et al., 2015; Kaplan and Ewers, 2015; Katoh et al., 2015; Klamecka et al., 2015; Nieuwenhuizen et al., 2015; Platonova et al., 2015a,b; Roebroek et al., 2015; Shin et al., 2015; Wedeking et al., 2015; Berry et al., 2016; Chamma et al., 2016, 2017; Drees et al., 2016; Gross et al., 2016; Harper et al., 2016; Joensuu et al., 2016; Künzl et al., 2016; Moutel et al., 2016; Tang et al., 2016a,b; Teng et al., 2016; Wendel et al., 2016; Katrukha et al., 2017; Roder et al., 2017; Almuedo-Castillo et al., 2018; Buser et al., 2018; De Beer et al., 2018; Gadok et al., 2018; Klein et al., 2018; Pani and Goldstein, 2018; Temme et al., 2018; Buser and Spiess, 2019; Cramer et al., 2019; Ghosh et al., 2019; Mann et al., 2019; Osswald et al., 2019; Prole and Taylor, 2019; Seitz and Rizzoli, 2019; Sograte-Idrissi et al., 2019; Farrants et al., 2020; Joshi et al., 2020
Gp41 (HIV)	cDNA	LM	Lutje Hulsik et al., 2013; Boersma et al., 2019
H2A-H2B	cDNA	LM	Jullien et al., 2016
HER2	Protein	LM / CLEM	Kijanka et al., 2013; Rakovich et al., 2014; Zou et al., 2015; D’Hollander et al., 2017; Kijanka et al., 2017; De Beer et al., 2018; Ramos-Gomes et al., 2018; Martinez-Jothar et al., 2019; Debie et al., 2020
HIF-1α	Protein	LM	Groot et al., 2006
HIV-1	cDNA	LM / SR	Helma et al., 2012
Heterochromatin Protein 1α	cDNA or Protein	LM	Moutel et al., 2016
Homer1	cDNA or Protein	LM / SR	Dong et al., 2019
Human Neonatal Fc Receptor	Protein	LM	Andersen et al., 2013
Huntingtin	cDNA	LM	Southwell et al., 2008; Maiuri et al., 2017
IGFBP7	Protein	LM	Iqbal et al., 2010b; Tomanek et al., 2012
IRSp53	cDNA	LM	Dong et al., 2019
Lamin	cDNA or Protein	LM / SR	Rothbauer et al., 2006; Schmidthals et al., 2010; Roder et al., 2017; Klein et al., 2018
L-plastin	cDNA	LM	Delanote et al., 2010; De Clercq et al., 2013
LY-6C/6G	Protein	CLEM	Fang et al., 2018
Marburgvirus	Protein	LM	Darling et al., 2017; Sherwood and Hayhurst, 2019
MHC II	Protein	LM	Rashidian et al., 2015; Duarte et al., 2016; Cheloha et al., 2019
Mouse IgG	Protein	LM / SR	Pleiner et al., 2018
NFT2	cDNA	LM	Van Audenhove et al., 2013
NTA domain cortactin	cDNA	LM	Bertier et al., 2018
Nup35	Protein	SR	Ma et al., 2017
Nup37	Protein	SR	Ma et al., 2017
Nup85	Protein	LM / SR	Pleiner et al., 2015
Nup93	Protein	LM / SR	Pleiner et al., 2015; Göttfert et al., 2017
Nup98	Protein	LM / SR	Pleiner et al., 2015; Göttfert et al., 2017
Nup155	Protein	LM / SR	Pleiner et al., 2015
n-WASP	cDNA	LM	Hebbrecht et al., 2017
p53	cDNA or Protein	LM	Moutel et al., 2016
PARP1	cDNA	LM	Buchfellner et al., 2016
PCNA	cDNA	LM	Burgess et al., 2012; Panza et al., 2015; Schorpp et al., 2016
PepTag	cDNA	LM	Traenkle et al., 2020
PFR1	Protein	LM	Obishakin et al., 2014
POM121	Protein	SR	Ma et al., 2017
PSMA	Protein	LM	Fan et al., 2015
Rabbit IgG	Protein	LM / SR	Pleiner et al., 2018
RFP / mCherry	cDNA or Protein	LM / SR / CLEM	Ries et al., 2012; Bleck et al., 2014; Platonova et al., 2015a,b; Wedeking et al., 2015; Moutel et al., 2016; Teng et al., 2016; Ariotti et al., 2018; Buser et al., 2018; Buser and Spiess, 2019; Cramer et al., 2019; Prole and Taylor, 2019; Sograte-Idrissi et al., 2019
SAPAP2	cDNA	LM	Dong et al., 2019
NAP-25	Protein	LM / SR	Maidorn et al., 2019
Survivin	cDNA	LM	Beghein and Gettemans, 2017
Syntaxin 1A	Protein	LM / SR	Maidorn et al., 2019
Tau (phosphorylated)	Protein	LM	Li et al., 2016
Tubulin	cDNA or Protein	LM / SR	Olichon and Surrey, 2007; Mikhaylova et al., 2015; Moutel et al., 2016
VEGFR	cDNA	LM	Ahani et al., 2016
Vimentin	cDNA or Protein	LM / SR	Maier et al., 2015, 2016; Klein et al., 2018
VSG	Protein	LM	Stijlemans et al., 2004
Vsig4	Protein	LM	Zheng et al., 2019
Vγ9Vδ2-T cell	Protein	LM	De Bruin et al., 2016

*EM, electron microscopy; SR, super resolution fluorescence microscopy; CLEM, correlated LM/EM.*

### cDNA Delivery of Chromobodies for Intracellular Targeting

**FIGURE 2:**
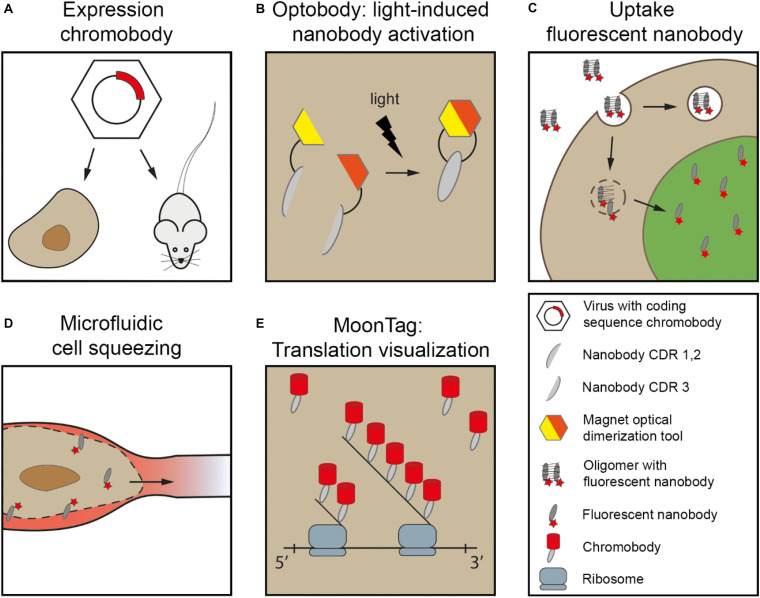
Nanobodies delivered for intracellular live-cell imaging. **(A)** Chromobodies cDNA loaded in e.g., adeno-associated viral particles (AAV). These viruses are used to infect cells in culture or in animals e.g., mouse. **(B)** Magnetic optical dimerization tool exists of two nMagHigh1 and pMagHigh1. Here, the nMagHigh1 is fused to nanobody fragment containing CDR1/2 and pMagHigh1 is fused to nanobody fragment containing CDR3. Upon stimulation of light, the magnetic tool paired together, resulting in restoring the nanobody. **(C)** Fluorescent nanobodies in e.g., oligomers can be taken up via endocytosis. Here, when endosomal rupture is induced, the free fluorescent nanobodies can bind to their target. **(D)** Microfluidic cell squeezing for temporary cell permeabilization. Purified fluorescent nanobodies are present in the medium, and diffuse into the accessible cytoplasm. **(E)** MoonTag: array of 24 peptide sequence repeat allows visualization of single molecules directly following translation by signal amplification. Chromobodies accumulate at the peptide chain. Major inspiration for this cartoon is from Roder et al. (2017), Klein et al. (2018), Boersma et al. (2019), Yu et al. (2019).

The first nanobody-based visualization of intracellular targets was achieved by the fusion to FPs (“chromobodies” Rothbauer et al., 2006; Table 1), being expressed in the target cells. Since then more nanobodies where developed targeting proteins inside cells (Table 2, delivered as cDNA). These intracellular chromobodies have been applied in small organisms like Danio rerio (Panza et al., 2015), Drosophila melanogaster (Harmansa et al., 2017), Caenorhabditis elegans (Pani and Goldstein, 2018) and Toxoplasma gondii (Tosetti et al., 2019) allowing live-cell and intravital microscopy. Intracellular expression of chromobodies in mice was successfully achieved by infecting the mice with adeno-associated viral particles containing the coding sequence of the chromobody (Figure 2A) (Wegner et al., 2017). Importantly, these chromobody approaches opens the opportunity for intravital imaging of endogenous proteins.

Next to defining protein localization, chromobodies can also relay functional changes in cells by fusing the nanobody to a fluorescent sensor for e.g., Ca^2+^ or pH (Prole and Taylor, 2019). For instance, anti-GFP nanobodies are fused to a Ca^2+^ sensor targeting GFP labeled mitochondria (Zhao et al., 2011). The nanobody facilitates the Ca^2+^ sensor to be in close proximity of the mitochondria to allow for Ca^2+^ dependent fluorescence readout. This results in the imaging of the local Ca^2+^ concentrations upon different stimuli. In the same study, anti-GFP nanobodies were conjugated to a SNAP-tag, a 20 kDa protein, modified from the human DNA repair protein O^6^-alkylguanine-DNA alkyltransferase (Keppler et al., 2003). The SNAP-tag is used to recruit a chemical dye, which facilitates live-cell imaging for Chromophore-Assisted Light Inactivation (CALI) (Jacobson et al., 2008): laser-induced subcellular destruction of a protein of interest. Thus nanobodies allow precise molecular targeting and enable analysis of the function of biomolecules, as well as precise protein manipulation with CALI allowing a direct cause/consequence study in living cells.

#### Fluorescence Signal From Unbound Chromobodies

Despite the success of chromobodies as intracellular probes, a disadvantage is their continued presence. Chromobodies fluoresce whether or not they bind to their target, as opposed to immunolabeling techniques that include multiple wash-out steps for unbound reagents or genetic fusions between target and FPs. To reduce the signal from non-bound chromobodies, conditionally stable chromobodies have been developed (Tang et al., 2016b). These modified anti-GFP chromobodies are instable and rapidly degraded by the proteasome. When stabilized, however, these chromobodies will bind their target and consequently will no longer be degraded. Indeed, engineering and application of conditionally stable anti-GFP chromobodies resulted in a reduced background fluorescence (Tang et al., 2016b). The mutations in the genetically modified nanobodies are highly conserved within nanobodies and therefore the switching from stable to instable nanobodies is generically applicable.

Non-targeted fluorescence can also be reduced by enhancer nanobodies (Roebroek et al., 2015). When the enhancer nanobodies bind with GFP, it increases the fluorescence and stability of the GFP. Enhancer nanobodies were also applied in a method to track single molecules in live-cell imaging (Ghosh et al., 2019). Here, an array of nanobodies is fused to the protein of interest, and expressed in cells with cytosolic monomeric GFP. Upon GFP binding to the array of nanobodies, the GFP molecules increase in fluorescent intensity, resulting in an increased signal-to-noise ratio. This binding can in the microscope be seen as a single dot, that represents a single protein of interest. In conclusion, the signal-to-noise ratio of chromobodies can be improved by degrading unbound chromobodies or by enhancing the fluorescence of bound FPs, which both will be beneficial in the detection of proteins.

#### Delocalization of Targeted Proteins

Modifying cellular systems, in any way, may of course result in altered biology (Schnell et al., 2012). Interactions between endogenous proteins and ectopically expressed chromobodies can potentially influence the localization and function of the protein of interest. Binding of nanobodies to their target during or post-translationally may disrupt proper protein folding, macromolecule organization and/or transport. Despite the often-highlighted benefits of nanobody-technology, like any other technique their use should be validated including the effect on protein localization. Artificial modifying localization of proteins can be used on purpose for targeted interference. Chromobodies targeting endogenous actin-binding proteins, like gelsolin and cortactin, led to a disturbed actin distribution (Van Audenhove et al., 2013; Hebbrecht et al., 2017; Wegner et al., 2017; Bertier et al., 2018) paralleled with a decrease in both the number of invadopodia as well as extracellular matrix degradation. These factors are important in cell migration (Wolf and Friedl, 2009). Thus, chromobodies-assisted protein modulation allows to study the contribution of specific proteins of biology, including cell migration or some of its consequences, like the delay of metastasis (Bertier et al., 2018).

#### Controlled Nanobody Activation

Spatiotemporal control of chromobody function is desirable in several assays. To initiate functionalized chromobodies in a controlled manner, chemogenetics or light stimuli can be applied to influence the binding capacity of the nanobody during and after synthesis. Such chemogenetic control employs ligand-modulated antibody fragments (LAMAs): a circular permutated bacterial dihydrofolate reductase (cpDHFR) linked to the nanobody is in such a conformation that it recognizes and binds to the nanobody target. In the presence of cell permeable DHFR inhibitors, the conformation changes precluding the antigen binding site of the nanobody binding the target, and thereby loss of association of nanobody and target. This process can be reversed to activate the nanobody binding (Farrants et al., 2020).

The light-dependent nanobody, termed photobody, uses a genetic photocaged tyrosine variant that results in the inactivation of the antigen-binding site (Arbely et al., 2012; Mootz et al., 2019). The photocaged tyrosine is photo-labile, and upon light induction (365 nm) the antigen-binding properties of the chromobodies are restored. Optobody, a second light-dependent tool, uses a split nanobody with a N-terminal fragment containing CDR1 and CDR2, and a C-terminal fragment containing CDR3 (Figure 2B; Yu et al., 2019). When both fragments are genetically fused to an optical-induced dimerization tool [MagHigh (Kawano et al., 2015)], the complete nanobody folds upon light stimulus and thereby forms the antigen-binding site. However, a generic position to split the nanobody is lacking, and thus for every different nanobody optimization and validation is needed. Overall, the activation of nanobodies using light or chemogenetic stimuli gives spatiotemporal control over the nanobodies, allowing precise subcellular modulation followed by direct readout of the biological consequences on targets studied.

### Purified Fluorescent Nanobodies Delivered for Live-Cell Imaging

Nanobodies can also be generated in cellular systems, subsequently purified and/or modified and then used in bioassays. Typically, these are secreted by mammalian cells or produced in high yields by bacteria (Harmsen and De Haard, 2007). After purification, the nanobody can for instance be coupled to chemical dyes (Beghein and Gettemans, 2017) to create fluorescent nanobodies (Tables 1, 2). Dyes suitable for super-resolution microscopy (SRM), i.e., LM beyond the diffraction limit resulting in typically 20 – 100 nm lateral resolution [reviewed in (Schermelleh et al., 2019)], will increase the resolution when using nanobodies compared to IgGs because of the smaller size of the reagents used, reducing the linkage error discussed above (Ries et al., 2012). Alternative to conjugation of purified nanobody with small fluorescent molecules, chromobodies can be expressed and purified. These chromobodies can then be directly used in fluorescent microscopy studies because they contain both the targeting module as well as the fluorescent module (Figure 1). The benefit of using cDNA encoding protein modules is the ease to switch target or color with molecular cloning tools. To employ these fluorescent nanobodies in live-cells different delivery mechanisms for extracellular or intracellular targets have been created.

#### Extracellular Targets

The extracellular domain of peripheral membrane proteins in cultured cells is well accessible to ectopic added reagents and therefore straightforward to target in live-cell imaging. Nanobodies that target extracellular receptors may trigger receptor specific endocytosis, which can be important for e.g., drug delivery. Endocytosis can be triggered via binding with the human epidermal growth factor receptor 2 (HER2). Anti-HER2 nanobodies (Kijanka et al., 2013) coupled to fluorescent, drug containing nanoparticles (Martinez-Jothar et al., 2019), were indeed able to trigger endocytosis. After the trigger, uptake and cell viability was visualized to examine the effect of the therapeutic nanoparticles. So, receptor mediated endocytosis can be activated using fluorescent nanobodies to study therapeutic agents coupled to the nanobodies.

Super resolution localization of GFP surface-exposed by cells has been achieved while studing dynamic changes at the plasma membrane: The glycosylphosphatidylinositol (GPI)-anchored GFP reporter was further probed with Alexa647-conjugated nanobodies to enable SRM based on the blinking of the Alexa-dye. This resulted in higher resolution imaging of dynamic changes and detection of protein enrichments in the plasma membrane (Ries et al., 2012; Virant et al., 2018). Indirect visualization enables newly displayed proteins at the plasma membrane. Here, all available antigens first are blocked by unconjugated nanobody. Upon exocytosis stimuli, at the plasma membrane, new extracellular exposed antigens can be detected with fluorescent nanobodies (Seitz and Rizzoli, 2019). This pulse-chase approach allows dynamic studies, e.g., protein turnover, of receptors and other cell surface proteins.

#### Intracellular Targets

The plasma membrane is a physical barrier for the nanobodies to target intracellular proteins in live-cells. Therefore, custom delivery methods are needed to target endogenous proteins in (living) cells without permeabilizing the plasma membrane. If nanobody expression is not an option because it first needs chemical modification or the concentration should be well-controlled, a purified nanobody may be delivered to cells. Fluorescent nanobodies can enter cells via endocytosis, when they formed non-covalent complexes with oligomers (Figure 2C; Roder et al., 2017) or they undergo lipid-based protein transfection (Oba and Tanaka, 2012; Virant et al., 2018). After endocytosis, the nanobodies need to escape the endosomal system and the formed complexes need to be degraded. However, using this strategy one has to take into account that the efficiency of endosomal escape is low (Stewart et al., 2016) and the nanobodies in the endosomes are already fluorescent, resulting in localized labeling of the endosomal system.

To prevent cellular uptake via endocytosis, cell-permeable nanobodies were generated by the addition of a cyclic cell-penetrating peptide [cCPP; (Herce et al., 2017)]. cCPPs are arginine-rich peptides that facilitates direct penetration of the plasma membrane to enter directly into the cytoplasm, independent of endocytosis. The efficiency of the labeling is expected to be increased because endosomal escape after endocytic uptake is omitted. Another advantage of using the cell-penetrating peptide is the ability to co-transport recombinant proteins, e.g., GFP, inside the cells, when both are bound to the nanobody. Although the efficiency of this co-transport was low, this cell-permeable nanobody can be further explored to serve as a drug delivery vehicle. A major disadvantage of the cCPPs however, is the strong tendency to accumulate in the nucleolus.

Generating temporary permeable plasma membranes is another approach to artificially deliver cargo inside cells. Different methods to temporary permeabilize the membrane have been developed: (i) Electroporation to deliver nanobodies linked to fluorescent quantum dots (QDs) into cells (Shi et al., 2018). The QDs were used for single particle visualization of intercellular transport by targeting kinesin motor proteins (Katrukha et al., 2017); (ii) Artificial plasma membrane channels that allow chromobody delivery into cells can be formed by bacterial Streptolysin O (Teng et al., 2016). Unbound nanobodies are removed during rinsing and the channels are closed upon switching to a recovery medium; (iii) Photoporation in which a laser-induced transfection enables the delivery of the nanobodies intracellular, and when these are fluorescently labeled, these can be directly detected (Hebbrecht et al., 2020). (iv) Another method to induce temporary damage to the plasma membrane is cell squeezing through the small capillaries of a microfluidic system resulting in fragility of the plasma membrane (Figure 2D; Klein et al., 2018). While the cells are squeezed, extracellular proteins can diffuse into the cells. When cells leave the small capillary the plasma membrane recovers to its normal state. Obviously, the effect of any temporary permeabilization approach used to enable nanobodies entrance into cells, is that endogenous molecules might diffuse out or targets may delocalize.

### General Applicable Peptide Tags

Genetic fusion with FPs cDNA is the widely used technique for protein visualization in living systems, but sometimes smaller peptide tags are preferred. Currently, there are no nanobodies available against common generic peptide tags (Muyldermans et al., 2001; Stijlemans et al., 2004; De Genst et al., 2006; Braun et al., 2016). Hence, three new small tags have been developed along with their respective targeting nanobodies. (i) The BC2-tag is a 12 amino acid peptide sequence originating from β-catenin (Braun et al., 2016; Virant et al., 2018), but the nanobody does not recognize endogenous β-catenin making it fairly specific for the tag only. (ii) The ALFA-tag (13 amino acids) forms an α-helix and is naturally absent in eukaryotes (Gotzke et al., 2019), also making it a specific target for its nanobody, which also counts for the (iii) Pep-Tag (15 amino acids) (Traenkle et al., 2020).

An array of peptides fused to the protein of interest can amplify the fluorescent signal, and thus increase the signal-to-noise ratio. A peptide-repeat called MoonTag (Boersma et al., 2019) was created to visualize active translation (Figure 2E). Here, an array of a 15 amino acid peptide sequence was added N-terminal of the protein of interest. The newly formed peptide chain forms a docking site for chromobodies. The MoonTag can be combined with the SunTag; an intracellular expressed single-chain variable fragment (scFV; Tanenbaum et al., 2014). Combining the two tags will allow visualization of different reading frames within a single mRNA or can be used to amplify the signal from different targets. Given the mechanism of probing the target with a cDNA encoded peptide repeat, making use of the same antibodies, this is a highly versatile enhancer system.

## Nanobodies in Electron Microscopy

While resolution of targets in LM is improved by using nanobodies because of a reduced linkage error compared to IgG targeting, the ultrastructural remains unexplored. In electron microscopy (EM), the ultrastructure is revealed, but localizing the protein of interest within this structure also requires probes. EM-visualization of targets benefits from the nanobody-technology because the probe is small and thus penetrates better. Therefore, milder permeabilization is needed, better preserving the ultrastructure. Moreover, the small size improves the resolution compared to traditional immuno-EM because the target and identifiable tag are in close proximity. Also in EM proteins can be specifically identified using genetically encoded or affinity-based probes. Genetically encoded probes may be based on peroxidases that creates black precipitates in the presence of diaminobenzidine (DAB) and H_2_O_2_. Affinity-based probes include electron dense nanoparticles like nanogold and QDs (De Boer et al., 2015). Of course the genetically encoded probes form good candidates to use in conjunction with nanobodies as a multi-modular probe for EM studies.

### Intracellular Nanobody Expression

**FIGURE 3:**
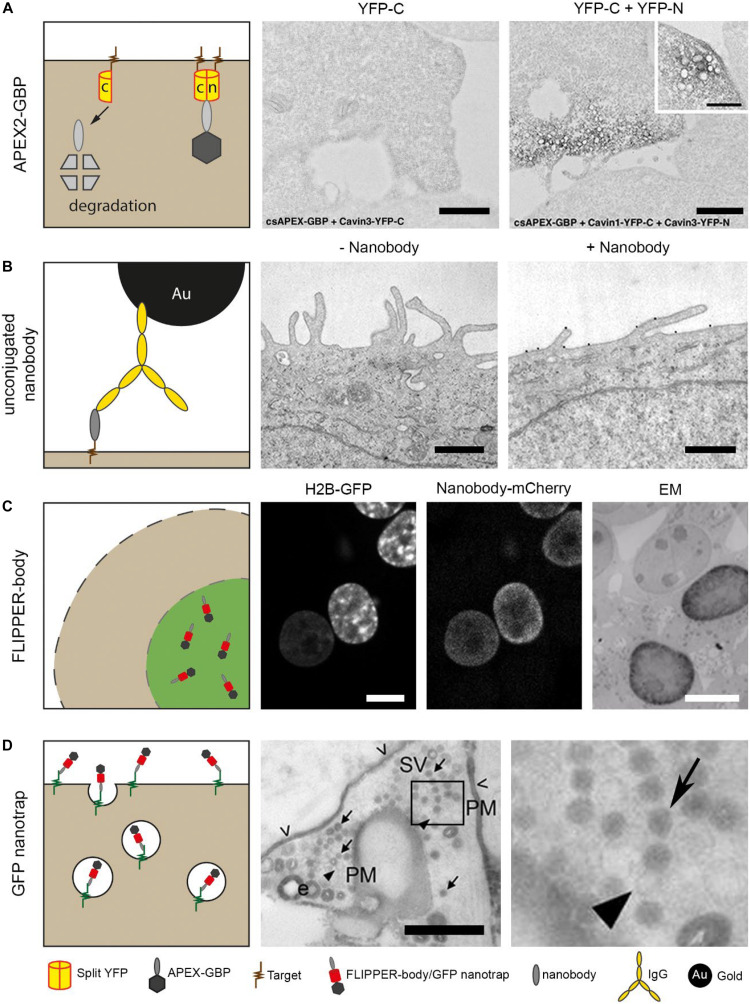
Nanobody technology for EM. **(A)** Cells were expressing only C-terminus YFP or both C- and N-terminus YFP. The cells also expressed a conditionally stable anti-YFP, for proteasomal degradation of unbound probe. The nanobodies were genetically fused with peroxidase, APEX2, for EM detection. Note that only black precipitation is visible in cells expressing C-terminus YFP and N-terminus YFP. This confirmed the degradation of the nanobody with the peroxidase. Bars: 1 μm. **(B)** Purified nanobodies were used as primary antigen binding protein to reduce the distance between label and antigen. Nanobodies can be detected using an anti-VhH IgG conjugated to gold for EM visualization. Bars: 0.5 μm. **(C)** Cells express H2B-GFP from Figure 1A, and are permeabilized after fixation followed by labeling with anti-GFP FLIPPER-bodies. Note the colocalization in LM and the dark, positive nucleus in EM versus an unlabeled nucleus. Bars: 10 μm. **(D)** Neuronal cells expressing pHluorin on the plasma membrane. Added anti-GFP nanobodies bind to the pHluorin, and after a pulse stimulation, synaptic vesicles are formed. In EM, a population of unlabeled and labeled synaptic vesicles is detected. Arrows indicate synaptic vesicles, arrow heads indicate unstained vesicles and open arrow heads indicate residual staining (PM, plasma membrane, SV, Synaptic vesicle). Bar: 0.5 μm. The images in **(A–D)** have been reproduced from the following studies (Joensuu et al., 2016; Kijanka et al., 2017; Ariotti et al., 2018; De Beer et al., 2018), all of which have been published under a Creative Commons Attribution License.

Intracellular nanobodies fused with soybean ascorbate peroxidase (APEX2) (Lam et al., 2015) can target GFP or mCherry to add an electron dense mark to the protein of interest at EM level (Ariotti et al., 2015, 2017). This APEX2-nanobody can be applied as general CLEM (correlated LM/EM) probe because many cells and small organisms have already been engineered to express GFP or mCherry. Using conditionally stable nanobodies (section Nanobodies In Light Microscopy), unbound APEX2-nanobody is degraded and thereby improve EM detection of the target proteins (Ariotti et al., 2018). The conditionally stable APEX2-nanobody enables studying protein-protein interactions by making use of splitFPs as target (Figure 3A; Ghosh et al., 2000; Hu and Kerppola, 2003). Here, a protein of interest and its potential interacting protein are genetically fused with different segment of e.g., GFP. Anti-GFP APEX2-nanobody can only bind to the folded GFP, representing the interaction between the two proteins of interest. When GFP is absent, the conditionally stable APEX2-nanobodies are degraded. Another alternative to study protein-protein interactions would be by using nanobodies fused to splitHRP or splitAPEX2 (Martell et al., 2016; Han et al., 2019). Two distinct nanobodies recognizing proteins within close proximity will facilitate the refolding of the peroxidase, which will result in DAB precipitates at that location in the sample. The split peroxidase thus reduces the signal of unbound nanobodies, leading to a more conclusive picture. In conclusion, where chromobodies allow dynamic studies of endogenous proteins, APEX2-nanobodies enable high resolution analysis in the ultrastructural context.

#### Nanobodies as Immunolabeling Reagent

Purified nanobodies can be applied for affinity-based immunolabeling, and thereby replace conventional IgGs. Immunolabeling with unconjugated nanobodies was used to label HER2 in breast cancer cells, followed by a secondary anti-nanobody IgG conjugated with nanogold (Figure 3B; Kijanka et al., 2017). Although this already improved the resolution, it could benefit more by replacing the IgG a direct labeling method using conjugated nanobodies.

### CLEM and Nanobodies

Reagents used in CLEM that are readily detectable both in the FLM and EM, like nanogold or QDs, are relatively large and/or bulky in comparison to 15–30 kDa range FPs or even smaller fluorescent dyes like FITC. These inorganic nanoparticles whether or not conjugated to nanobodies, will have limited penetration (Giepmans et al., 2005; Schnell et al., 2012). Therefore, we developed a completely protein-based probe, called “FLIPPER”-body (Kuipers et al., 2015; De Beer et al., 2018) with (i) a nanobody as targeting module; (ii) a FP for LM analysis; and (iii) a horseradish peroxidase (HRP) for EM analysis. The FLIPPER-bodies were produced by mammalian cells applied as immunoreagent to label e.g., intracellular GFP (Figure 3C). Other targets were generated by simple molecular cloning interchanging the modules of the probe. FLIPPER-bodies improve penetration due to its size and flexibility comparted to IgGs-targeted nanoparticles and thus lead to a better target detection while maintaining reasonable ultrastructure.

In conventional EM glutaraldehyde is used to fix the samples for ultrastructural preservation (Schnell et al., 2012). However, cells fixed with paraformaldehyde become permeable for small molecules like e.g., small fluorescent nanobodies (Fang et al., 2018). This labeling without adding permeabilization reagents method was further developed to stain intracellular targets in fixed mouse tissue slices. When targeting an intracellular target with both nanobody and IgG, the nanobody penetrated up to 100 μm into a brain tissue slice, while the IgGs only stained the surface of the slice. Subsequently, the same cells were imaged with EM and ultrastructural details were well preserved due to glutaraldehyde fixation after performing the nanobody labeling. So, detergent treatment is not required when using small nanobody probes and thus cellular structure is better preserved. The technique needs to mature further to establish the ratio of positives and false negatives to determine if the recognition of the targets is generically reliable. Moreover, the accessibility of targets within organelles, like mitochondria or nucleus, remains to be studied further.

#### Nanobodies for Live-Cell Imaging and EM

Alternatively to expressing nanobody-based probes in the cellular system of interest, they also can first be purified and then applied to cells. An elegant example of this approach has been used to visualize the formation of synaptic vesicles in living cells (Joensuu et al., 2016). Ultrastructural localization of newly formed synaptic vesicles can be examined using peroxidase-fused nanobodies. Vesicle-associated membrane protein 2 (VAMP2) mediates the formation of synaptic vesicles and is expressed at the plasma membrane of neuronal cells. Anti-GFP nanobodies were applied to target pHluorin, a GFP derivative, fused with VAMP2 (Figure 3D). Upon K^+^ stimulation, pHluorin-VAMP2 with bound nanobody is endocytosed, and new synaptic vesicles are formed. After EM preparation, the newly formed synaptic vesicles were stained black, as a result of the peroxidase attached to the nanobody, whereas older synaptic vesicles remained unstained. Thus, the endocytic route of specific proteins of interest can be visualized.

Purified nanobodies, fused to a FP and a peroxidase, were used to analyze the retrograde transport system in live-cells (Buser and Spiess, 2019) or within ultrastructural context (Buser et al., 2018) to study the transport from the cell surface to the Golgi complex. Cells with GFP-modified cycling reporter proteins at the plasma membrane captured the nanobodies extracellular and transported them in the cells. CLEM revealed the dynamic behavior of different cycling reporter proteins, and showed the ultrastructural localization of the reporter proteins. Overall, these nanobody-based CLEM probes can reveal the localization of plasma membrane proteins and visualize their dynamics, together with the ultrastructural context.

### Nanobodies in Cryo-EM

Structural analysis of proteins at atomic resolution is achieved by cryo-EM [reviewed in (Kühlbrandt, 2014; Egelman, 2016; Cheng, 2018)]. However different conformations of proteins may be present, hindering the structural determination of specific states. Here, nanobodies can help to stabilize the proteins into a certain conformational state [reviewed in (Uchański et al., 2020)], especially as the targeting module in rigid chimeras termed megabodies. These stabilizing nanobody-chimeras recently have been used to solve the type A γ-aminobutyric (GABA_*A*_) structure in membranes-like structures in presence and absence of natural ligands and antagonists (Laverty et al., 2019; Masiulis et al., 2019). Using the megabodies, no longer the target proteins themselves need to be engineered and thus structure on the endogenous proteins is being revealed. Likely, nanobody technology will further contribute to structural biology on many other target proteins by facilitating single conformational protein states and better understand the dynamic regulation of biomolecules by their ligands without directly altering the proteins at study.

## Nanobodies for Microscopy: To Date a Great Potential But Such a Limited Use

Nanobodies have clear benefits over conventional IgGs antibodies or genetically encoded probes. Nanobodies (i) have a small diameter resulting in better resolution and better penetration; (ii) can visualize endogenous proteins in live-cell imaging; (iii) are encoded by a one cDNA, which enables easy molecular cloning; (iv) allow researchers to create and produce custom multi-modal probes. Potential artifacts specific for nanobody technology in microscopy include signal from chromobodies independent of target binding and modified localization of the protein target, as detailed above.

Nanobodies are still limited used in research, mainly due to the limitation in the availability of nanobodies for general targets compared to IgGs and researchers are not aware of the possibilities of nanobody in microscopy. Here, we aim to increase the visibility of opportunities and benefits highlighted in the main text, but also exemplified by the successful new insights and new nanobody-based reagents by many (Table 2). Other ways to improve the use of nanobodies in research are open access sharing of nanobodies cDNA to allow researchers to create and manipulate their own labeling reagent and to increase the nanobody database of general targets. Currently, most nanobodies are generated via *Camelidae* immunization, a route that may be a (seemingly) hurdle for scientists. However, these days the generation of nanobodies is both economically and time-wise competitive with generation of newly synthesized rabbit IgG against specific targets. The novice user obviously will benefit of expertise and accessibility to the needed infrastructure by more experienced users mentioned throughout this review. In addition, a fast method based on a yeast display platform to select nanobodies *in vitro* has been established (McMahon et al., 2018).

### From Current State to Future Outlook: Nanobodies Towards a Common Technique

Protein identification in microscopy has been greatly aided by immunolabeling using IgGs as well as the application of genetically encoded tags. Nanobody technology, being explored for approximately 25 years is a great additional tool. Like IgGs, endogenous proteins can be easily studied without direct modification. Moreover, the single domain properties and therefore the easy use as a module in other genetically encoded probes allow freedom of use of tags and targeting module. In line with SRM techniques, nanobodies have the added benefit over IgGs that they hardly provide distance between target and label (linkage error). The major limitation in the use of nanobodies in general is the sparse availability of high affinity nanobodies for specific targets. Increasing the availability for different targets using immunization and/or micro-organism-based libraries will increase the variation in the decade to come. When more targets can be studied with nanobodies these will become a common tool in the lab and share the top-3 podium with genetically encoded tags as well as IgGs since they share benefits of both approaches to identify and visualize targets of interest in dynamic systems and with high precision.

## Author Contributions

MB performed the experiment and made the figures. MB and BG wrote the manuscript. Both authors contributed to the article and approved the submitted version.

## Conflict of Interest

The authors declare that the research was conducted in the absence of any commercial or financial relationships that could be construed as a potential conflict of interest.
